# Sex Bias in Gut Microbiome Transmission in Newly Paired Marmosets (Callithrix jacchus)

**DOI:** 10.1128/mSystems.00910-19

**Published:** 2020-03-24

**Authors:** Lifeng Zhu, Jonathan B. Clayton, Mallory J. Suhr Van Haute, Qinnan Yang, Haley R. Hassenstab, Aaryn C. Mustoe, Dan Knights, Andrew K. Benson, Jeffrey A. French

**Affiliations:** a Department of Biology, University of Nebraska at Omaha, Omaha, Nebraska, USA; b Nebraska Food for Health Center, University of Nebraska—Lincoln, Lincoln, Nebraska, USA; c Department of Psychology, University of Nebraska at Omaha, Omaha, Nebraska, USA; d Department of Food Science and Technology, University of Nebraska—Lincoln, Lincoln, Nebraska, USA; e BioTechnology Institute, College of Biological Sciences, University of Minnesota, Minneapolis, Minnesota, USA; f Department of Computer Science and Engineering, University of Minnesota, Minneapolis, Minnesota, USA; Duke University School of Medicine

**Keywords:** social behavior, common marmosets, pair-bond formation, longitudinal sampling, social transmission, sex bias, gut microbiome transmission

## Abstract

Social behavior can alter the microbiome composition via transmission among social partners, but there have been few controlled experimental studies of gut microbiome transmission among social partners in primates. We collected longitudinal fecal samples from eight unrelated male-female pairs of marmoset monkeys prior to pairing and for 8 weeks following pairing. We then sequenced 16S rRNA to characterize the changes in the gut microbiome that resulted from the pairing. Marmoset pairs had a higher similarity in gut microbiome communities after pairing than before pairing. We discovered sex differences in the degrees of change in gut microbiome communities following pairing. Specifically, the gut microbiome communities in males exhibited greater dissimilarity from the prepairing stage (baseline) than the gut microbiome communities in females. Conversely, females showed a gradual stabilization in the rate of the gut microbiome community turnover. Importantly, we found that the male fecal samples harbored more female-source gut microbes after pairing, especially early in pairing (paired test, *P < *0.05), possibly linked to sex bias in the frequencies of social behavior. From this controlled study, we report for the first time that pair-living primates undergo significant changes in gut microbiome during pairing and that females transmit more microbes to their partners than males do. The potential biases influencing which microbes are transmitted on the basis of sex and whether they are due to sex biases in other behavioral or physiological features need to be widely investigated in other nonhuman primates and humans in the future.

**IMPORTANCE** In this controlled study, we collected longitudinal fecal samples from 16 male and female marmoset monkeys for 2 weeks prior to and for 8 weeks after pairing in male-female dyads. We report for the first time that marmoset monkeys undergo significant changes to the gut microbiome following pairing and that these changes are sex-biased; i.e., females transmit more microbes to their social partners than males do. Marmosets exhibit pair bonding behavior such as spatial proximity, physical contact, and grooming, and sex biases in these behavioral patterns may contribute to the observed sex bias in social transmission of gut microbiomes.

## Abstract (precis)

In this controlled study, we collected longitudinal fecal samples from 16 male and female marmoset monkeys for 2 weeks prior to and for 8 weeks after pairing in male-female dyads. We report for the first time that marmoset monkeys undergo significant changes to the gut microbiome following pairing and that these changes are sex-biased; i.e., females transmit more microbes to their social partners than males do. Marmosets exhibit pair bonding behavior such as spatial proximity, physical contact, and grooming, and sex biases in these behavioral patterns may contribute to the observed sex bias in social transmission of gut microbiomes.

## INTRODUCTION

Animal gut microbiomes (GM) play an important role in the host’s nutrition, immune system function, and overall health (1, 2), and animal diet and phylogeny are two of the main factors influencing variation the gut microbial composition and function (3, 4). There is a growing interest in the notion that behavioral processes, including common group membership and social interaction patterns, also serve as significant predictors of the similarities and differences in the organization of gut microbial communities (5). Social transmission of gut microbes has been demonstrated or implicated in species ranging from invertebrates to humans, including bumblebees (6), barn swallows (7), zebra finch (8), giraffe (9), ponies (10), baboons (11), sifakas (12), and humans and human-companion dogs (13). Interestingly, one study on the zebra finch cloaca microbiome finds a high and unidirectional rate of microbiomal transmission from males to females, but not the converse, likely as a consequence of cloacal deposition of sperm by males (8). Further, one study showed that close social relationships among human adults correlate with human gut microbiome composition; in families living together, marital spouses had more similar microbiota and more bacterial taxa in common than sibling pairs (14, 15).

Those previous studies demonstrated the importance of horizontal transmission of microbiome constituents as a consequence of social interaction patterns. However, little is known about the temporal and longitudinal changes in the gut microbiome during the establishment of socially interacting individuals. Longitudinal prospective studies can offer important information about temporal trends in gut microbiome communities in a number of contexts (16, 17), including infant development (18), treatment with antibiotics (19), and migration (20). Thus, there are basic open questions regarding the relationship between social behavior and gut microbiome transmission (GMT) during initial cohabitation with a social partner. (i) What are the longitudinal changes in the gut microbiome community after pairing? (ii) Does a directional bias exist in the gut microbiome transmission between the partners?

A small proportion of nonhuman primates exhibit socially monogamous mating systems with social, sexual, and affiliative contact among adults typically limited to a single male-female pair (21–23). The close social network and daily iterative interactions between socially monogamous males and females is an ideal model to explore gut microbiome transmission in paired individuals. Here, we investigated these questions in the marmoset monkey (Callithrix jacchus), a New World primate that displays many of the social components of monogamy, including high rates of affiliation (grooming and huddling), shared parental care, and joint defense of territories (24). Upon pairing with a new mate, marmosets (*Callithrix* spp.) display elevated rates of social and sexual interactions, including social grooming, genital investigation, social approach, and mounts and copulations (25–27). These behavioral patterns could facilitate the transfer of microbial taxa between pairmates. In the first few weeks of pairing, there is also a sex bias in the directionality of social behavior, with males exhibiting higher rates than females of anogenital investigation, initiation of grooming, and initiation of spatial proximity in the first weeks after pairing relative to later phases of the social relationship (25–29).

We examined changes in the gut microbiome community and a potential directional sex bias in gut microbiome transmission using longitudinal fecal samples from eight pairs of common marmosets during the establishment of new adult male-female pairs. Baseline fecal samples were collected during a 2-week period prior to pairing (PRE), during which marmosets resided with an opposite-sex partner or in a family group. Marmosets were then rehoused in a new enclosure with a previously unfamiliar and unrelated marmoset of the opposite sex. Fecal samples were collected in the postpairing phase (POST) for an 8-week period, during which diet and other environmental variables remained constant.

## RESULTS

### Gut microbiome organization in captive common marmosets.

We obtained 16S rRNA MiSeq sequence reads from 240 fecal samples from 16 adult marmosets across an approximately 2.5-month period. Demographic information on the subjects can be found in Table S1 in the supplemental material. Fifty-three samples were collected in the 2-week PRE phase (range of samples per individual, 2 to 5), and 187 samples were collected in the POST phase (range of samples per individual, 6 to 15) (Table S1). To decrease the sequencing depth bias, we performed rarefaction (5,000 reads per sample) on these 240 fecal samples. The marmoset microbiome is characterized by high proportions of *Firmicutes* (39.1%), *Bacteroidetes* (29.2%), *Actinobacteria* (26.9%), and *Proteobacteria* (4.0%) based on the fecal samples from the 16 adult individuals in this study. The predominant families in the gut microbiome included *Bifidobacteriaceae*, *Veillonellaceae*, *Bacteroidaceae*, *Acidaminococcaceae*, *Prevotellaceae*, *Lachnospiraceae*, *Coriobacteriaceae*, *Enterobacteriaceae*, *Porphyromonadaceae*, and *Succinivibrionaceae*, accounting for 97% of bacterial abundance in the overall data set (Table S2).

10.1128/mSystems.00910-19.5 TABLE S1 Subject and sample information. Download Table S1, DOCX file, 0.01 MB. Copyright © 2020 Zhu et al. 2020 Zhu et al. This content is distributed under the terms of the Creative Commons Attribution 4.0 International license.

10.1128/mSystems.00910-19.6 TABLE S2 The ten most abundant families in the common marmoset gut microbiome. p_, phylum. Download Table S2, DOCX file, 0.03 MB. Copyright © 2020 Zhu et al. 2020 Zhu et al. This content is distributed under the terms of the Creative Commons Attribution 4.0 International license.

### Changes in the gut microbiome diversity and community during pairing.

**FIG 1:**
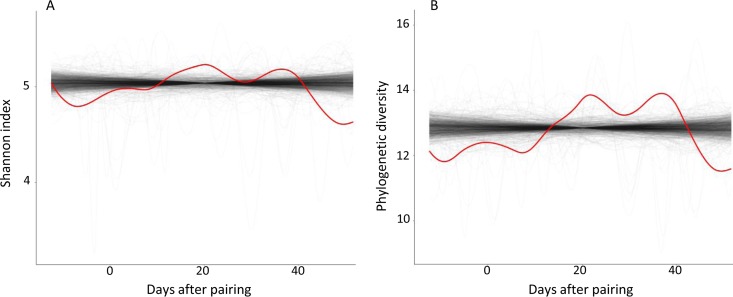
The results from the trendyspliner function showed that the permuted data formed a zero-change distribution from which the real data (red line) of the Shannon index (A) and phylogenetic diversity (B) were significantly distinct (99 permutations, *P* < 0.05). The translucent lines represent the permuted splines under the total of 99 random permutations. The red lines represent the group spline (alpha diversity).

We used the trendyspliner tool (17) to test whether there were temporal changes in alpha diversity after pairing. The results from the trendyspliner function showed that the permuted data formed a zero-change distribution from which the real data (red line) of Shannon index (Fig. 1A) and phylogenetic diversity (Fig. 1B) were significantly distinct (*P < *0.05). However, the plot from the permuspliner test showed that the alpha diversity values in females were not significantly different from those in males (99 permutations; permuspliner test, *P > *0.05) (see Fig. S1 in the supplemental material).

10.1128/mSystems.00910-19.1 FIG S1 The permuspliner plots exhibit the alpha diversity over time in the female (group spline in red) and the male (group spline in blue, 99 permutations, *P* > 0.05). (Left) Shannon index. (Right) Phylogenetic diversity. The translucent lines represent the number of permuted splines under the total of 99 random permutations. Download FIG S1, DOCX file, 1.5 MB. Copyright © 2020 Zhu et al. 2020 Zhu et al. This content is distributed under the terms of the Creative Commons Attribution 4.0 International license.

**FIG 2:**
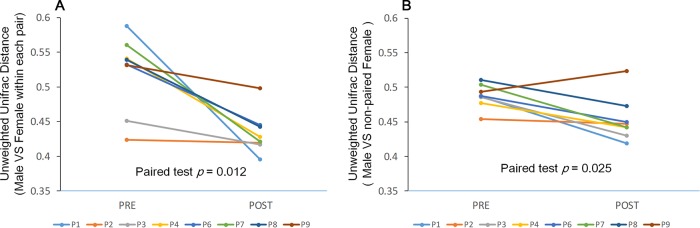
Average beta diversity (unweighted UniFrac distances) between PRE and POST. (A) UniFrac distances within the pair significantly decreased after pairing (Wilcoxon test, *P = *0.012). (B) UniFrac distances also decreased among randomly paired males and females that were not cohabiting in the same enclosure (Wilcoxon test, *P = *0.025), indicating a consensus change in the gut microbiome community across pairs. P1, P2, P3, P4, P6, P7, P8, and P9, pairs 1, 2, 3, 4, 6, 7, 8, and 9, respectively.

**FIG 3:**
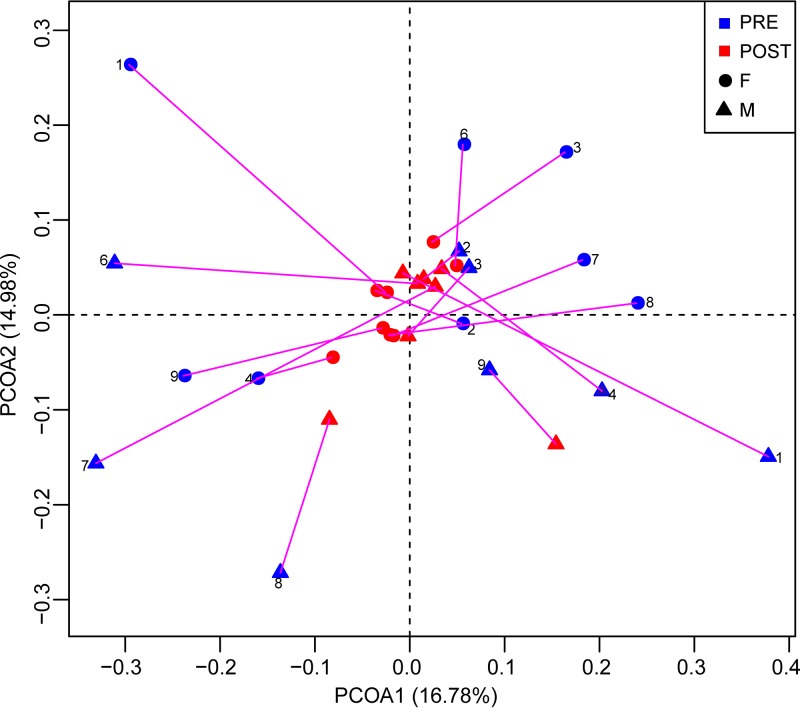
PCoA plots using unweighted UniFrac distances for PRE and POST fecal samples. The number inside the plots represents the pair number. Lines connect individual marmosets in the two stages of the study. F, females; M, males.

We further assessed gut microbiota similarity within each pair using unweighted UniFrac distances. UniFrac scores within the pairs significantly decreased in the POST phase compared to the PRE stage, indicating an increase in gut microbiome similarity among male-female pairs (Fig. 2A; Wilcoxon test, *P < *0.05). We also compared UniFrac scores from PRE to POST for randomly selected, noncohabiting male-female pairs. UniFrac scores in this comparison also showed a significant reduction across stages (Fig. 2B; Wilcoxon test, *P < *0.05), indicating an increase in the gut microbiome similarity and a convergence in microbiome communities. Unweighted UniFrac distances, displayed as principal-coordinate analysis (PCoA) plots (Fig. 3), also revealed (i) differences in the gut microbiome communities among the individuals in the PRE and POST stages, (ii) low similarity in the gut microbiome communities among male-female pairs in the PRE phase, and (iii) high similarity in the gut microbiome communities among the males and females in the POST phase. Thus, these findings suggest that pairing is associated with increases in gut microbiota community similarity and convergence both within pairs and across pairs.

**FIG 4:**
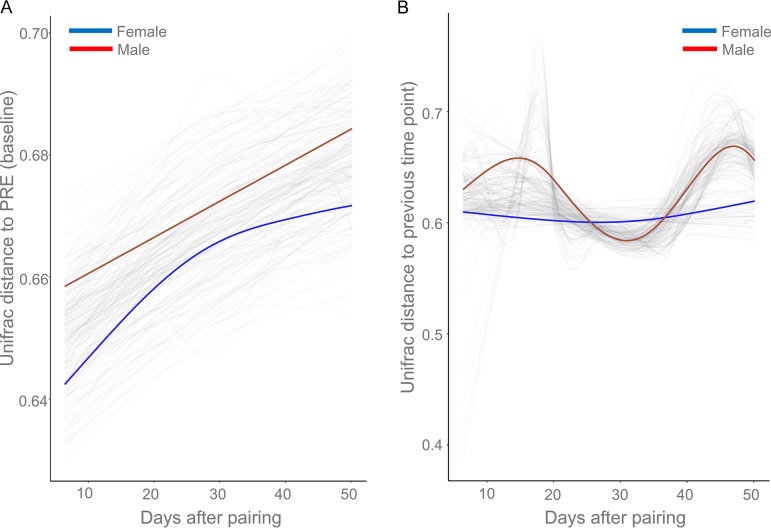
The permuspliner plots exhibited that males (red line) and females (blue line) differed in the longitudinal change in unweighted UniFrac distances from the PRE stage for each individual (A) and between successive samples collected from the same individual after pairing (B). The translucent lines represent the permuted splines with 99 random permutations. The lines represent the group splines for males (red) and females (blue).

We further tracked the volatility in beta diversity (unweighted UniFrac distances) after pairing using the “first distances” method (16). We assessed how an individual gut microbiome community differed from the PRE (baseline)/POST over time. The results (Fig. 4A) showed that (i) the gut microbiome community in both males and females diverged from the communities present in the PRE stage over time and (ii) the gut microbiome in males exhibited greater dissimilarity from the PRE stage than did the gut microbiome in females. The differences between the sexes in UniFrac scores were significant in the early stages of pairing (Fig. S2; Mann-Whitney test, *P < *0.05). Moreover, we also investigated the changes in beta diversity (unweighted UniFrac distances) between successive samples from the same individual after pairing (Fig. 4B). Males showed a dramatic shift in the early and end stages of pairing, and females showed a gradual stabilization in the rate of gut microbiome community turnover. These findings indicate that males and females exhibited different rates of gut microbiome community turnover after pairing. Overall, the results from the first distances analysis demonstrated putative sex differences in the effects of pairing on the gut microbiome community.

10.1128/mSystems.00910-19.2 FIG S2 Result of the sliding spliner function displaying the *P* value at each specified interval derived from the distribution of points from individuals’ smoothed splines. The dotted line indicates *P = *0.05. There was a significant difference between the proportions of gut microbiome transmission in the female and the male in the early stage of pairing. Download FIG S2, DOCX file, 0.1 MB. Copyright © 2020 Zhu et al. 2020 Zhu et al. This content is distributed under the terms of the Creative Commons Attribution 4.0 International license.

### Sex bias in gut microbiome transmission during pairing.

In the first few weeks after pairing, males typically show higher rates of sniffing, grooming, and genital investigation than females (24–28). Therefore, we evaluated whether there were sex biases in the transmission of microbiota among newly paired marmosets at the finest taxonomic scale level available (enumeration of precise amplicon sequence variants). We used SourceTracker (30), a Bayesian approach using source communities, to identify sources, directly estimate their proportions in the sink samples, and model the uncertainty about known and unknown sources. Given the difference in the gut microbiome between sexes at the PRE stage, we established two sources within each pair: the gut microbiome identified in the female fecal samples and the gut microbiome from the male fecal samples. Each fecal sample collected after the pairing stage was treated as a sink sample. Thus, we obtained the source proportion for each fecal sample in the POST phase.

**FIG 5:**
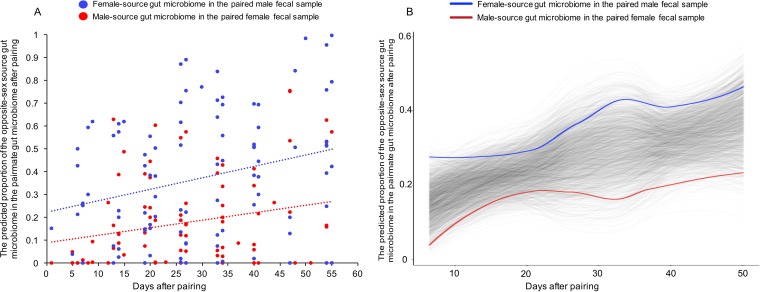
SourceTracker (30) analysis using the finest taxonomic level (amplicon sequence variants) revealed a sex bias in the gut microbiome between the female and male after transmission. (A) The proportion of the putative female-source gut microbiome in the male gut microbiome per pair (blue) and the proportion of the putative male-source gut microbiome in the female gut microbiome within the pair (red) after pairing. The lines represent the linear trend lines. (B) Results of permuspliner analysis performed using the data in panel A show the putative sex bias in gut microbiome transmission between the female and male over time. The translucent lines represent the permuted splines derived from 99 random permutations. The red line represents the group spline of the putative male-source gut microbiome in the female gut microbiome within the pair. The blue line represents the putative female-source gut microbiome in the male gut microbiome within the pair.

The putative gut microbiome transmission from the pairmate increased over time postpairing, especially for males (Fig. 5). For example, in the first 2 weeks of pairing, six of eight females in the study showed no evidence of colonization by microbes predicted to be from their male partner, while all of the males had some proportion of microbes that were identified as “female source” (Fig. 5A). Moreover, based on the permutation analysis, the putative gut microbiome transmission from the pairmate increased significantly over time after pairing (permuspliner, *P < *0.05). The proportions of the gut microbiome transmission (from the opposite sex) to each sex were significantly different in the first 2 weeks after pairing (Fig. S3; Mann-Whitney test, *P < *0.05). Thus, our data suggest a significant horizontal transmission of components of the gut microbiome as a consequence of cohabitation and social interaction in marmosets, with males acquiring proportionately more taxa from their female partners than the converse.

10.1128/mSystems.00910-19.3 FIG S3 The permuspliner plots of the contribution by GMT1 (belonging to *Phascolarctobacterium*) representing gut microbiome transmission between the female and male after pairing. Data highlighted in blue represent the contribution of GMT1 from the female to the male within the pair. Data highlighted in red represent the contribution of GMT1 from the male to the female within the pair. The translucent lines represent the number of permuted splines under the total of 99 random permutations. Download FIG S3, DOCX file, 0.1 MB. Copyright © 2020 Zhu et al. 2020 Zhu et al. This content is distributed under the terms of the Creative Commons Attribution 4.0 International license.

After estimating the overall proportion of the gut microbiome transmitted between partners, we investigated the pattern of microbiome transmission at the finest taxonomic scale level (using amplicon sequence variants). Most of the taxa identified as being shared between pairmates came from 14 genera, including *Firmicutes* (e.g., *Phascolarctobacterium*, *Pribacterium*, *Megasphaera*, *Megamonas*, and the *Lachnospiraceae* FE2018 group), *Actinobacteria* (e.g., *Olsenella*, *Collinsella*, and *Bifidobacterium*), *Bacteroidetes* (*Paraprevotella*, *Prevotella 9*, *Bacteroides*, and *Alloprevotella*), and *Proteobacteria* (e.g., *Escherichia-Shigella* and *Hafnia-Obesumbacterium*). The mean proportion of contributions from six gut microbiome transmission taxa (GMT) was significantly higher in female-to-male transmission than in male-to-female transmission (Table 1) (Wilcoxon test, *P = *0.05). For example, GMT1 (*Phascolarctobacterium*) showed the highest contribution to the sex-biased transmission pattern of females to males (Fig. S3; permuspliner *P = *0.033).

**TABLE 1:**
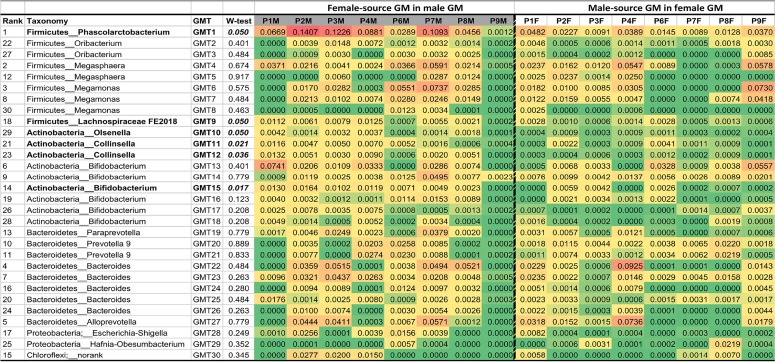
The proportion of the predicted contribution of the microbiome (at the finest taxonomic scale using amplicon sequence variants) to gut microbiome transmission between males and females^*a*^

a Rank is based on the mean proportion of the predicted contribution of the microbiome to the gut microbiome transmission between the paired sex partners after pairing. GM, gut microbiome; GMT, putative gut microbiome transmission; W-test, paired Wilcoxon test; P1M, the male in pair 1; P1F, the female in pair 1. Taxa in bold indicate a significant difference in female-to-male and male-to-female microbiome transmission. Red cells indicate a high proportion of contribution to GMT, and green cells indicate a low contribution to GMT. Each number corresponding to the presence of female-source GM in the male GM part represents the mean proportion of the contribution by the specific microbiome in the total female-source gut microbiome present in the male gut microbiome. Each number corresponding to the presence of male-source GM in the female GM part represents the mean proportion of the contribution by the specific microbiome in the total male-source gut microbiome present in the female gut microbiome. Taxonomy, the genus information for each GMT.

10.1128/mSystems.00910-19.7 TABLE S3 Significant changes in the abundance of the gut microbial genera after pairing. The text in parentheses presents a detailed classification of each genus. p_, phylum; f_, family. The order of genera is based on the linear discriminant analysis (LDA) values (from large to small). Download Table S3, DOCX file, 0.1 MB. Copyright © 2020 Zhu et al. 2020 Zhu et al. This content is distributed under the terms of the Creative Commons Attribution 4.0 International license.

### Longitudinal microbiome variation during pairing.

**FIG 6:**
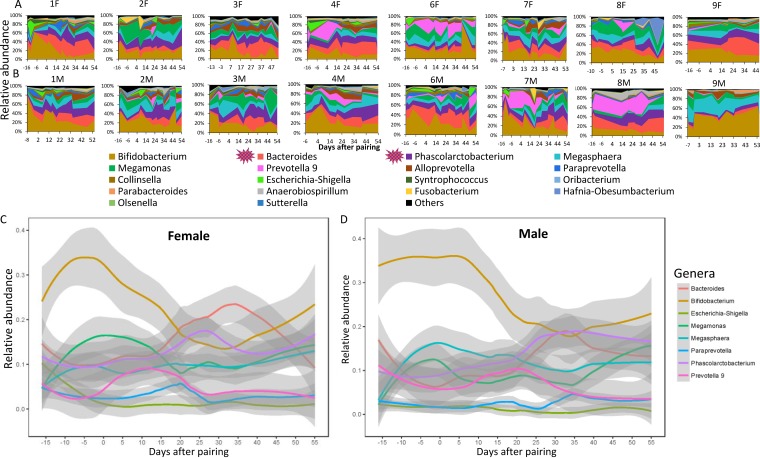
Longitudinal microbiome variation after pairing. (A and B) Taxonomic area charts of the relative abundance of dominant genera in females (A) and males (B). The two spiny red-and-purple symbols that appear in the key below panel B indicate main contributions of genera in gut microbiome transmission. Each study animal is represented by “P” followed by a numeral and “M” or “F”; e.g., P1M represents the male in pair 1 and P1F represents the female in pair 1. (C and D) Spline plot of mean abundance changes in the top eight gut microbial genera in the females (C) and males (D) in this longitudinal experiment.

In addition to pair-specific convergence in microbial communities, we also noted common patterns of change in the gut microbiome across all pairs and both sexes. These changes included a disruption to the gut microbiome immediately after pairing and expansion of *Phascolarctobacterium* and *Bacteroides* (Fig. 6A and B). We further studied the dynamics of changes in the abundance of the eight most common genera (accounting for 83% of the bacterial abundance in the overall data set) across time using spline-plotting methods (17). We found decreases in the abundance of *Bifidobacterium* and increases in *Bacteroides*, *Phascolarctobacterium*, and *Anaerobiospirillum* in female and male fecal samples after pairing (Fig. 6C and D; see also Fig. S4). We then estimated the changes in the genera after pairing using Lefse (linear discriminant analysis effect size) (31). We found the abundance of five bacteria (*Phascolarctobacterium*, *Alloprevotella*, *Anaerobiospirillum*, *Sutterella*, and *Coprobacter*) significantly increased in both female and male fecal samples after pairing (Table S3). In contrast, the abundance of *Bifidobacterium*, *Escherichia-Shigella*, and *Weissella* significantly decreased in both female and male fecal samples after pairing (Table S3). Therefore, this controlled study showed that some of the changes in overall microbiome composition following pairing with a new social partner were common across individuals.

10.1128/mSystems.00910-19.4 FIG S4 Changes in abundance of *Anaerobiospirillum* over time in female (red) and male (blue) fecal samples determined by permuspliner analysis. The translucent lines represent the number of permuted splines under the total of 99 random permutations. Download FIG S4, DOCX file, 1.9 MB. Copyright © 2020 Zhu et al. 2020 Zhu et al. This content is distributed under the terms of the Creative Commons Attribution 4.0 International license.

## DISCUSSION

This research reports the first longitudinal study on the effects of cohabitation and the establishment of a close social relationship with an opposite-sex partner on the gut microbiome in primates, using the pair-living marmoset Callithrix jacchus as a model. Gut microbiome similarity within the paired marmosets increased over time after cohabitation. Moreover, we found the following sex differences in the effects of pairing on the gut microbiome. (i) Male gut microbiome communities exhibited greater UniFrac distances from the PRE stage than did females. (ii) Males displayed more-dramatic shifts between successive samples after pairing. (iii) Females showed a more gradual stabilization in the rate of gut microbiome community turnover. This sex difference in the volatility turnover may be related to a sex bias in gut microbiome transmission after pairing; males harbored more taxa identified as female source over time than the converse.

Previous studies have demonstrated that social behavior (e.g., grooming or close spatial proximity) can lead to similarity in the structures of the gut microbiomes (11, 12, 32), but most of those studies were performed in wild populations. While those studies have considerable ecological validity, it is difficult to separate the effects of social interactions on microbiome similarity from those of commonalities in shared dietary intake or exposure to local environmental microbes. In contrast, our work was conducted under conditions of constant and carefully controlled environments, and marmosets were fed identical diets throughout the study. Our data show that cohabitation with an opposite-sex partner, with whom there is significant social and sexual contact immediately after pairing, strongly impacted the gut microbiome community by increasing gut microbiota similarity within the pair.

We identified sex biases in the gut microbiome transmission, in that the males harbored more female-source gut microbes, especially in the first weeks after pairing. In humans, marital spouses have higher gut microbiome similarities than siblings or nonrelated individuals (14). The data showing a female-to-male sex bias in horizontal microbe transmission presented here is consistent with behavioral evidence indicating that, in the first week or two after pairing, males exhibit higher rates of partner grooming than females and also engage in higher rates of anogenital investigation of the partner than females (25–29). The sex differences in investigatory behavior and contact with the partner could account for a more efficient transfer of multiple components of the microbiome from females to males. Based on the detailed longitudinal analysis of the putatively transmitted gut microbiome, we observed that the marmosets experienced convergence in the gut microbiome within pairs and across pairs after pairing, with a high contribution of *Phascolarctobacterium* and *Bacteroides* in gut microbiome transmission. The more “invasive” microbiome transmission happening in most of the eight pairs after pairing in this study, such as transmission of *Phascolarctobacterium* (belonging to class *Selenomonadales* in *Firmicutes*), led to differences in the gut microbiome community between PRE and POST samples and to enhanced similarity within the pair or between the pairs. Interestingly, a previous study on wild baboons also observed that the social partners shared not only more-similar gut microbiome communities but also similar abundances of some phylogenetically related microbial taxa (e.g., *Selenomonadales*) (11). However, the reason(s) for their high invasiveness and their potential function is not yet known.

Although our experiment introduced several careful controls, there were several limitations. For example, both pairing of male-females specifically and simple cohousing of any familiar or nonfamiliar conspecifics under the same environment could influence the gut microbiome community. These two factors (social transmission and shared environmental factors) are not independent of each other. We speculate that the sex bias in gut microbiome transmission after pairing may be related to social behavior, but in the future, one interesting approach might be to directly assess whether the magnitude of microbiome transmission is proportionate to differences in the frequencies of specific social behaviors. Additional longitudinal cohousing experiments on same-sex pairs (e.g., female-female or male-male pairs) would also serve as a valuable control to test whether there are other underlying reasons for sex biases in social transmission outside behavioral output. However, this is not easy to test in marmosets as unrelated same-sex pairs are often unaffiliative toward each other and can display high rates of aggression.

We observed a profound decrease in *Bifidobacterium* at the onset of pairing in both females and males. These changes may have been associated with high stress/arousal at the beginning of pairing, which has been reported previously in marmoset pairs (27). During the initial phase of the pairing, the animals have elevated levels of glucocorticoids (cortisol), with a return to baseline levels afterward (28, 33). A negative correlation between free urinary cortisol levels and *Bifidobacterium* has been demonstrated in humans and rats (34). Future experiments will aim to specifically address the relationship between postpairing increases in glucocorticoids and changes in the microbiome.

### Conclusion.

Formation and maintenance of cooperative and reciprocal social relationships are important behavioral outputs for a variety of species. An important and specific example of this reciprocal relationship is that between adult males and females in pair-living species (35, 36). Examples include cichlid fishes (37), the majority of avian species (38), and some mammals (36). The establishment of a socially monogamous relationship between partners is associated with many social factors, such as spatial proximity, physical contact, and social interactions (39). Our findings revealed for the first time that a pair-living primate, the common marmoset, undergoes profound changes to the gut microbiome, with a directional sex bias in the gut microbiome during early pair-bonding formation. Gut microbiome transition began within days after pairing. This report offers novel insight into the relevant transmission patterns within a dynamic and reciprocal social network revealing that long-term social interactions are accompanied by a sex bias in the social transmission of microbiomes. These findings raise the possibility that sex-specific patterns of transmission of potentially deleterious and/or protective microbiome communities are relevant to the overall microbiome composition of individuals, and, consequently, that these socially derived changes in microbiomes may potentially impact the overall health status of individuals.

## MATERIALS AND METHODS

### Subjects.

We examined the gut microbiota in eight adult common marmoset pairs (eight females and eight males; ages, 1.5 to 7.5 years) (Table S1). These individuals were housed at the Callitrichid Research Center at the University of Nebraska at Omaha. Diets were composed of a commercial marmoset diet (Zupreeml Science Diet), *Tenebrio* larvae, scrambled eggs, fruits (red apple and cantaloupe), and gum Arabic (Mazuri). The husbandry protocols are summarized in reference 26.

This study was performed following the guidelines of the University of Nebraska Medical Center and the University of Nebraska at Omaha Institutional Animal Care and Use Committee. The protocol was approved by the University of Nebraska Medical Center/University of Nebraska at Omaha Institutional Animal Care and Use Committee (protocol 16-104).

### Sample collection (longitudinal study).

Fresh fecal samples (Table S1) were collected from marmosets in sterilized aluminum pans immediately after the light-on phase of the photoperiod. Samples were snap-frozen in liquid nitrogen and stored at −80°C. We collected fresh fecal samples across 16 adult individuals across an approximately 2.5-month period (prepairing stage, about 2 weeks; postpairing stage, about 2 months). Demographic information on the pairs can be found in Table S1.

### DNA extraction and MiSeq sequencing.

Total DNA was extracted using a BioSprint 96 One-For-All kit (384) (Qiagen), and PCR amplification of the V4 region of the 16S rRNA gene was performed using V4f (GTGCCAGCMGCCGCGGTAA) and V4r (GGACTACHVGGGTWTCTAAT) primers as described previously (40). Reaction products were purified and sequenced on the MiSeq platform (Illumina) (41).

### Basic analysis of 16S rRNA gene data.

We trimmed the raw data sets using strict trim parameters and deleted chimeras to obtain a final clean set of 5,000 reads (normalized) per sample. QIIME v1.9.0 was used to identify OTUs (operational taxonomic units) based on 97% similarity (42). The taxon was annotated by the use of a SILVA132 16s database. QIIME 1.9 was used to calculate the phylogenetic diversity and unweighted UniFrac distances (42).

### Volatility in alpha diversity over time.

SplinectomeR is designed to summarize data in longitudinal studies through smoothing splines (17). Here, we used the trendyspliner function in SplinectomeR to evaluate whether the alpha diversity increased overall in a nonzero direction over time (17). The group spline was fitted to our real data (alpha diversity), and the linear baseline was established from the start point for the group in this study, including the PRE stage. The area between the group spline and the baseline was estimated as the nonzero change. Therefore, if the alpha diversity increased over time, the areas would be large (17). The null distribution was generated by permutation of the time series for each individual. From the random distribution of areas, generated by the repeated permutations (99 permutations), two-sided *P* values were determined by comparisons to the observed values (17). Moreover, we also used the permuspliner function in SplinectomeR to evaluate whether the differences in the alpha diversities in the female and male that occurred over time were greater than would be expected by random chance (17). The loss spline was fitted to the data in a total time series. The observed group distances between the male and female alpha diversities were calculated over time, and null distributions over the random between-group distances were generated by the repeated permutations (99 permutations). Then, the empirical *P* value was measured by comparison to the observed distances between the female and male alpha diversities (17).

### Changes in beta diversity after pairing.

First, we compared levels of microbial similarity in the two stages of the study (PRE versus POST) using the unweighted UniFrac distances by collapsing the time points to a single averaged point to indicate whether the pairing increased the gut microbiome similarity within the pair. We arranged the data in blocks from the period after the pairing stage, given the possible behavioral changes caused by the establishment of a pair bond (25, 29, 43, 44) and the possible gut microbiome changes. Here, 14-day averages were calculated for each block. Thus, we obtained five blocks (PRE, block 1_14, block 15_28, block 29_42, and block 43_55). For each pair, we determined two single average points, one from the PRE stage and the other from the POST stage (days 29 and 55 after pairing). Wilcoxon paired tests were used to calculate the *P* value from these two groups (PRE versus POST). We also compared the distances between the male and a randomly selected female in the PRE and POST stages to determine whether pairing led to a common change in the gut microbiome community. In the PRE stage, the male per potential pair (i.e., each male that would be paired in the POST stage) gained seven mean distances compared with each nonpaired female (i.e., each female that would not be paired in the POST stage). In the POST stage, the male per pair gained seven mean distances compared with each nonpaired female. We then obtained the two single averaged points for the male in each pair, one from these seven distances in PRE and the other one from seven distances in POST. Wilcoxon paired tests were used to calculate the *P* value from these two stages (PRE versus POST).

Second, we used the q2-longitudinal package (16) and SplinectomeR (17) to test whether the beta diversities differed with respect to volatility between the males and females over time. This would provide a way of looking at the potential sex differences in the effects of pairing on the gut microbiome community. The q2-longitudinal package is available as a plugin in QIIME2 (45). For each animal, we calculated the unweighted UniFrac distances between each sample in the POST stage and the samples in the PRE stage. Then, we used the permuspliner function in SplinectomeR to test whether the distances corresponding to the female and male followed similar trends over time (17). We used the sliding spliner function in SplinectomeR to test whether the two groups (males and females) were significantly different at any point in time after pairing (99 permutations).

Finally, for each individual in the POST stage, we calculated the unweighted UniFrac distances between samples from successive time points using the first distances method in the q2-longitudinal package (16). This method was also used to assess how the rate of change differed over time. In gaining this longitudinal distance matrix (including individual information, sex information, and information representing the days after pairing) for all individuals in the POST stage, we used the permuspliner function in SplinectomeR to test whether the distances of the female and male followed the more different trend over time (17). We used the sliding spliner function in SplinectomeR to test whether the two groups (the female and male) were significantly different at any point in time after pairing (99 permutations). In addition, the PCoA plots for PRE and POST individuals were generated by QIIME using the average group unweighted UniFrac distances.

### Putative gut microbiome transmission between males and females.

The proportion of putative gut microbiome transmission within each pair was predicted by SourceTracker (30). SourceTracker (30) is a Bayesian approach using source communities to identify sources, directly estimate their proportions in the sink samples, and model the uncertainty about known and unknown sources. In order to do a better test for the putative gut microbiome transmission, we applied deblur (46) to produce the microbiome unit table (the input file for SourceTracker) at the finest taxonomic scale (amplicon sequence variants). In each pair, there were two gut microbiome sources; one was from the PRE female fecal samples, and the other was from the PRE male fecal samples. Also, there were two gut microbiome sinks per pair: POST female fecal samples and POST male fecal samples. Thus, for each pair, we quantified the proportion of female source in each male sink feces sample and the proportion of male source in each female sink feces sample. Then, we combined the information from each of the POST samples to produce the longitudinal table on gut microbiome transmission (including the proportion of source, sex information, individual information, and information on days after pairing). We used the permuspliner function in SplinectomeR to test whether there was a sex bias in the gut microbiome transmission between the female and male over time (17). We used the sliding spliner function in SplinectomeR to test whether the two groups (males and females) were significantly different at any point in time after pairing (99 permutations). SourceTracker (30) also provided the proportion of the contribution of each microbiome unit in gut microbiome transmission. Thus, we treated these microbiome units making a high level of contribution as the putative gut microbiome transmission (GMT). We used the permuspliner function in SplinectomeR to test whether the sex bias in the contribution of some unique GMTs changed over time.

### Changes in abundance of the gut microbiome during pairing.

We performed Lefse (linear discriminant analysis effect size) analysis to detect differences in abundant bacteria among groups (31). We used ggplot2 to plot the loss spline for these significantly different microbiome groups over time.

### Data availability.

Sequencing data have been submitted to NCBI with BioProject accession number PRJNA607180.
