# Supplementary material for: Sex Bias in Gut Microbiome Transmission in Newly Paired Marmosets (Callithrix jacchus)
Source: mSystems. 2020 Mar 24;5(2):e00910-19. doi: 10.1128/mSystems.00910-19 (PMC7093826; doi:10.1128/mSystems.00910-19)
Supplement: TABLE S1 [file mSystems.00910-19-st001.docx]

| **Animal** | **Pair ID** | **Sex** | **DOB** | **PRE Condition^1^** | **n^2^ (PRE/POST)** |
| --- | --- | --- | --- | --- | --- |
| ART | 1 | F | 2-Jul-09 | Pair | 4/12 |
| TAN | 1 | M | 19-Feb-14 | Pair | 2/12 |
| NYL | 2 | F | 28-Jul-10 | Pair | 3/11 |
| WOL | 2 | M | 28-Sep-14 | Family | 3/14 |
| LII | 3 | F | 26-Aug-10 | Pair | 3/13 |
| XMA | 3 | M | 28-Sep-14 | Family | 4/11 |
| ATH | 4 | F | 7-May-10 | Pair | 3/11 |
| YOS | 4 | M | 19-Feb-15 | Family | 2/11 |
| ELI | 6 | F | 24-Jul-15 | Family | 3/15 |
| DAC | 6 | M | 8-Oct-10 | Pair | 4/15 |
| ZEL | 7 | F | 19-Feb-15 | Family | 4/12 |
| JAX | 7 | M | 9-May-10 | Pair | 5/14 |
| URS | 8 | F | 5-Apr-14 | Pair | 4/10 |
| FAB | 8 | M | 15-Mar-09 | Pair | 3/9 |
| VEL | 9 | F | 7-Jun-09 | Pair | 2/6 |
| ODI | 9 | M | 22-Apr-10 | Pair | 4/11 |

^1^Social environment during the PRE stage of the study. Pair = housed with opposite-sex marmoset. Family = adult offspring housed in family group

^2^n = number of fecal samples collected in each stage of the study.
