# Supplementary material for: Sex Bias in Gut Microbiome Transmission in Newly Paired Marmosets (Callithrix jacchus)
Source: mSystems. 2020 Mar 24;5(2):e00910-19. doi: 10.1128/mSystems.00910-19 (PMC7093826; doi:10.1128/mSystems.00910-19)
Supplement: TABLE S2 [file mSystems.00910-19-st002.docx]

| **Family** | **Mean** | **SE** |
| --- | --- | --- |
| Bifidobacteriaceae (p_Actinobacteria) | 0.238 | 0.0096 |
| Veillonellaceae (p_Firmicutes) | 0.211 | 0.0082 |
| Bacteroidaceae (p_Bacteroidetes) | 0.141 | 0.0066 |
| Acidaminococcaceae (p_Firmicutes) | 0.137 | 0.0050 |
| Prevotellaceae (p_Bacteroidetes) | 0.132 | 0.0067 |
| Lachnospiraceae (p_Firmicutes) | 0.037 | 0.0017 |
| Coriobacteriaceae (p_Actinobacteria) | 0.031 | 0.0017 |
| Enterobacteriaceae (p_Proteobacteria) | 0.025 | 0.0039 |
| Porphyromonadaceae (p_Bacteroidetes) | 0.013 | 0.0008 |
| Succinivibrionaceae (p_Proteobacteria) | 0.009 | 0.0013 |
