# Supplementary figures and images for: Sex Bias in Gut Microbiome Transmission in Newly Paired Marmosets (Callithrix jacchus)
Source: mSystems. 2020 Mar 24;5(2):e00910-19. doi: 10.1128/mSystems.00910-19 (PMC7093826; doi:10.1128/mSystems.00910-19)

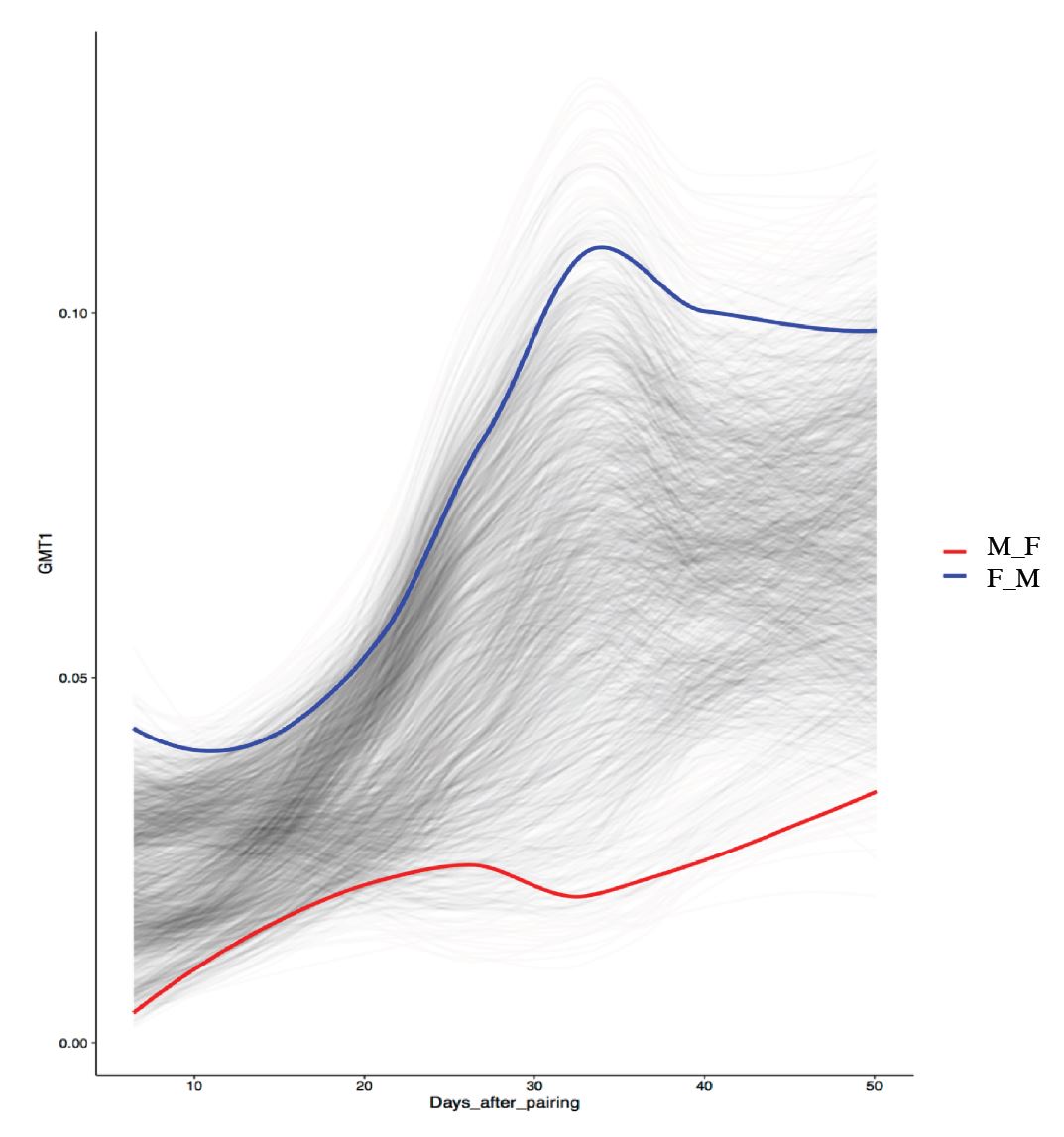

Supplement: FIG S3 [file mSystems.00910-19-sf003.docx]
