# Supplementary material for: Sex Bias in Gut Microbiome Transmission in Newly Paired Marmosets (Callithrix jacchus)
Source: mSystems. 2020 Mar 24;5(2):e00910-19. doi: 10.1128/mSystems.00910-19 (PMC7093826; doi:10.1128/mSystems.00910-19)
Supplement: TABLE S3 [file mSystems.00910-19-st003.docx]

|  | **Significant increasing** |  | **Significant**  **decreasing** |  |
| --- | --- | --- | --- | --- |
| **Female** | *Bacteroides* | (p_Bacteroidetes\|f_Bacteroidaceae) | *Bifidobacterium* | (p_Actinobacteria\|f_Bifidobacteriaceae) |
|  | *Alloprevotella* | (p_Bacteroidetes\|f_Prevotellaceae) | *Escherichia-Shigella* | (p_Proteobacteria\|f_Enterobacteriaceae) |
|  | *Phascolarctobacterium* | (p_Firmicutes\|f_Acidaminococcaceae) | *Collinsella* | (p_Actinobacteria\|f_Coriobacteriaceae) |
|  | *Megasphaera* | (p_Firmicutes\|f_Veillonellaceae) | *Syntrophococcus* | (p_Firmicutes\|f_Lachnospiraceae) |
|  | *Sutterella* | (p_Proteobacteria\|f_Alcaligenaceae) | *Neisseria* | (p_Proteobacteria\|f_Neisseriaceae) |
|  | *Anaerobiospirillum* | (p_Proteobacteria\|f_Succinivibrionaceae) | *Olsenella* | (p_Actinobacteria\|f_Coriobacteriaceae) |
|  | *Coprobacter* | (p_Bacteroidetes\|f__Porphyromonadaceae) | *Blautia* | (p_Firmicutes\|f_Lachnospiraceae) |
|  | *Selenomonas 1* | (p_Firmicutes\|f_Veillonellaceae) | *[Eubacterium] rectale group* | (p_Firmicutes\|f_Lachnospiraceae) |
|  |  |  | *Weissella* | (p_Firmicutes\|f_Leuconostocacea) |
| **Male** | *Phascolarctobacterium* | (p_Firmicutes\|f_Acidaminococcaceae) | *Bifidobacterium* | (p_Actinobacteria\|f_Bifidobacteriaceae) |
|  | *Alloprevotella* | (p_Bacteroidetes\|f_Prevotellaceae) | *Hafnia-Obesumbacterium* | (p_Proteobacteria\|f_Enterobacteriaceae) |
|  | *Anaerobiospirillum* | (p_Proteobacteria\|f_Succinivibrionaceae) | *Anaerofustis* | (p_Firmicutes\|f_Eubacteriaceae) |
|  | *Sutterella* | (p_Proteobacteria\|f_Alcaligenaceae) | *Kineosporia* | (p_Actinobacteria\|f_Kineosporiaceae) |
|  | *Prevotella 1* | (p_Bacteroidetes\|f_Prevotellaceae) | *Escherichia-Shigella* | (p_Proteobacteria\|f_Enterobacteriaceae) |
|  | *Coprobacter* | (p_Bacteroidetes\|f__Porphyromonadaceae) | *Abiotrophia* | (p_Firmicutes\|f_Aerococcaceae) |
|  |  |  | *Actinobacillus* | (p_Proteobacteria\|f_Pasteurellaceae) |
|  |  |  | *Streptococcus* | (p_Firmicutes\|f_Streptococcaceae) |
|  |  |  | *Weissella* | (p_Firmicutes\|f_Leuconostocacea) |
|  |  |  | *Hungatella* | (p_Firmicutes\|f_Lachnospiraceae) |
|  |  |  | *Acinetobacter* | (p_Proteobacteria\|f_Moraxellaceae) |
