# Supplementary material for: Sex Bias in Gut Microbiome Transmission in Newly Paired Marmosets (Callithrix jacchus)
Source: mSystems. 2020 Mar 24;5(2):e00910-19. doi: 10.1128/mSystems.00910-19 (PMC7093826; doi:10.1128/mSystems.00910-19)
Supplement: Supplementary file 5 [file mSystems.00910-19-sf001.docx]

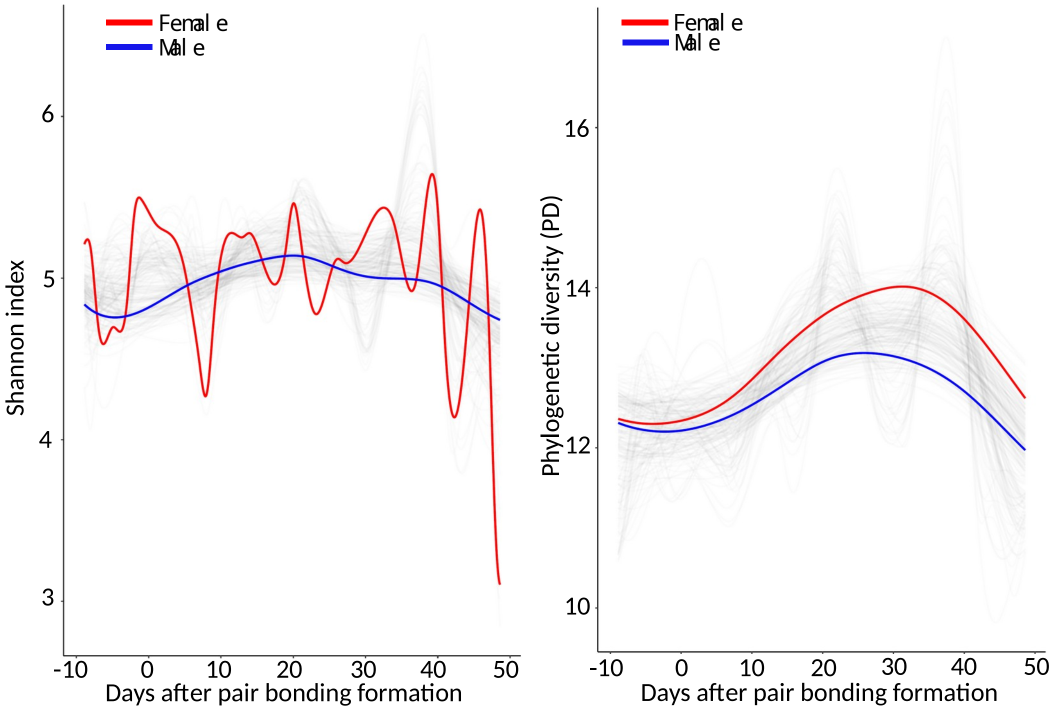
Female
Male
6
5
4
3
Shannon index
-10
0
10
20
30
40
50
Days after pair bonding formation
Female
Male
16
14
12
10
Phylogenetic diversity (PD)
-10
0
10
20
30
40
50
Days after pair bonding formation
