# Supplementary material for: Sex Bias in Gut Microbiome Transmission in Newly Paired Marmosets (Callithrix jacchus)
Source: mSystems. 2020 Mar 24;5(2):e00910-19. doi: 10.1128/mSystems.00910-19 (PMC7093826; doi:10.1128/mSystems.00910-19)
Supplement: Supplementary file 6 [file mSystems.00910-19-sf002.docx]

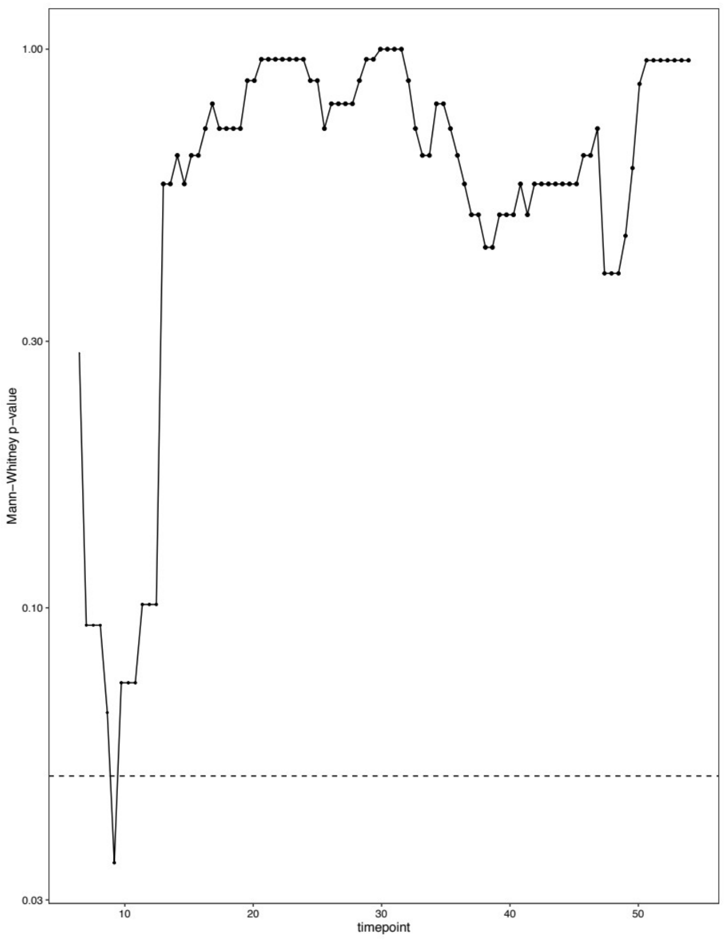
1.00
0.30
0.10
0.03
Mann−Whitney p−value
10
20
30
40
50
timepoint
