# Supplementary material for: Sex Bias in Gut Microbiome Transmission in Newly Paired Marmosets (Callithrix jacchus)
Source: mSystems. 2020 Mar 24;5(2):e00910-19. doi: 10.1128/mSystems.00910-19 (PMC7093826; doi:10.1128/mSystems.00910-19)
Supplement: Supplementary file 7 [file mSystems.00910-19-sf004.docx]

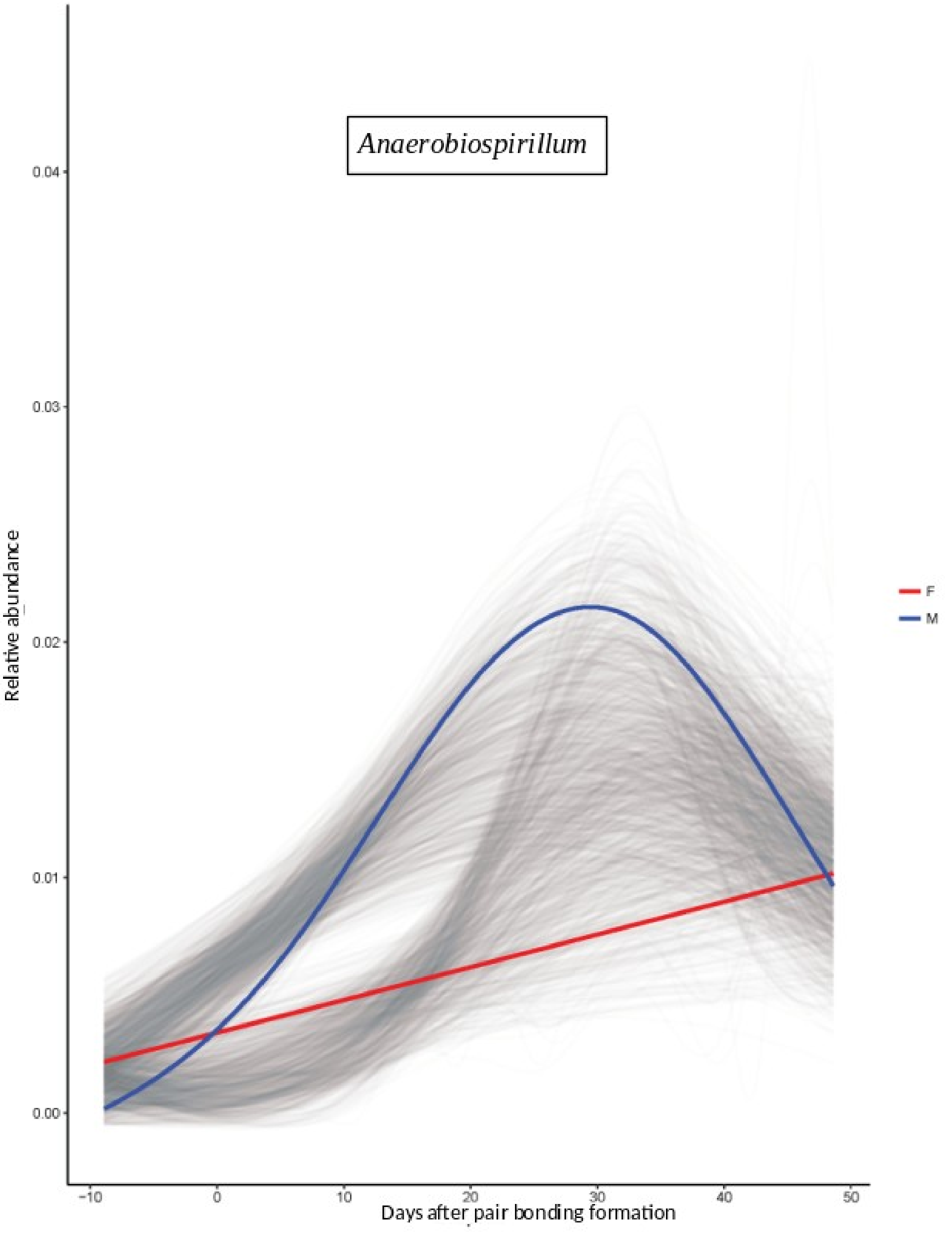
Anaerobiospirillum
0.04
0.03
0.02
0.01
0.00
Relative abundance
−10
0
10
20
30
40
50
Days after pair bonding formation
F
M
